# Distortions of the Feet

**Published:** 1875-01

**Authors:** 


### DISTORTIONS OF THE FEET.

We have given a good deal of attention to this subject lately, and have sought information from all possible sources. We have long felt, as has every experienced physician, that there was something radically wrong in the form of our boots and shoes. We never could comprehend why it should not be possible to decently clothe our feet, while at the same time preserving their symmetry, their elastic spring, and their comfort. We were unwilling to believe that painful corns were a necessity to the human race; that toe-nails ought to turn downwards, and cut the delicate flesh; that the toes *must* be piled upon each other; that the great toe should point towards the smaller toes, and so compel a bulging of the great toe joint; that painful spots had a natural right to exist upon the instep; in short, that pedal misery and ugliness were natural evils, ordained from the beginning of things, and not to be evaded. All these thoughts have haunted us for years. For years, too, we had been seeking something new, something better than the wretched old plan. We even searched the records and the models at the patent office in Washington, but without avail. Several parties claimed improvements upon the old style, but practical tests of their systems exhibited the fact that they had not hit the nail on the head. Their shoes were as uncomfortable and as unscientific as anybody's, some of them a trifle worse than the old kind.

After fairly giving up the chase, McComber and his lasts dawned upon us. The latter struck us favorably, but we hardly dared say a word in favor of them until we had worn the boots made on them. Even then we thought it might be only a lucky hit in our own case, and waited for further light. We talked with wearers of the new goods, and were rejoiced to find that all who had tried them were enthusiastic in their praise. We made the new last a good deal of a study. We saw that distorted feet began to assume a natural form very soon after commencing to wear the McComber shoes. We saw that badly crippled people soon began to walk easily and naturally. Then for the first time we felt safe in expressing ourselves favorably concerning the new system.

But we wanted more testimony. So we talked freely with Mr. Joel McComber, the inventor, of 21½ Spruce street, and asked him to give us the names of such of those for whom he had made shoes or lasts as he thought would be willing to be called upon. In this way we accumulated a great mass of testimony on the subject, all of it, we desire to say, of a favorable character. We conversed with Mr. Bristol, who manufactures ladies' goods on this last, at 8 Warren street, and here again much information, tending in the same direction, was secured.

Just before this number of our magazine was ready for the press, it occurred to us to call on Mr. F. Edwards, who has an immense boot and shoe store at 166 and 168 Atlantic avenue, Brooklyn, and get his views of the new system, as well as such expressions of opinion as he could remember to have been uttered in his presence by some of his customers. We found Mr. Edwards very enthusiastic on the subject of the McComber last. He has been a long time in the business, and feels that he has done more injury to the feet of humanity than he will probably ever atone for; but he sees that he is now on the right track, and the good which he is doing encourages him to persevere. He is doing his work in a most radical manner. He has taken advantage of the cold weather to warm up his large store with a few cart-loads of the old-fashioned lasts which he had on hand, and he speaks favorably of them - as fuel. He has supplied himself with the McComber lasts in all sizes and for all sorts of people, and no power can ever send him back to the abominable lasts used by others in the trade. He is very penitent over his past errors, and pledges himself to sin no more in that particular direction.

We asked him what his customers thought of the new last, and his good-natured countenance was all aglow as he recounted the remarks made to him by the intelligent wearers of goods made by his workmen on the McComber lasts. We wrote down the names of some well-known citizens, all of whom have worn these goods, and speak earnestly - some of them with deep feeling and enthusiasm—in favor of the new boots and shoes. Among the citizens of Brooklyn there are J. P. Underhill, Union Stores; Evelyn K. Johnson, 289 Hicks street; Mrs. Dr. Crane, 163 Clinton street; J. N. Freeman, 80 Hanson Place; the venerable Judge B. D. Silliman, 56 Clinton street; C. C. Adams, 138 Clinton street; Mrs. Gilkerson, 85 Montague street, and C. T. Dotter, 15 Tompkins Place. The same feelings are expressed by Dr. H. C. Vogell, Cold Spring, Long Island; John W. Tyler, harbor master; Mrs. T. H. Gillis, 115 East Seventieth street, and W. B. Ives, 98 Front street, all of New York city. There are quite a number of doctors and ministers on Mr. Edwards' books, whose testimony is given strongly in favor of the McComber system.

We asked Mr. E. if he was in the habit of receiving orders for goods by mail, and were told that a good many goods were shipped to distant parts in answer to mail orders. Lasts are made to fit the feet, and sent by mail or express, as are boots and shoes. Instructions are sent on application, so that the proper measure can be readily taken by any intelligent person.

Our little trip to Brooklyn in pursuit of information was very satisfactory to us. We found Mr. Edwards deeply impressed with the importance of the new method, fully aware of the fearful consequenees which attend the use of foot-clothing on the old plan, and with the brains and the financial ability to do a great deal towards remedying the horrible evil. We call on all intelligent men and women to aid him in the great work which he has assigned himself. He is a missionary in a very important sense, since he wages a very determined warfare against one of the direst physical evils of which the civilized world has knowledge—that of distortions of the feet, involving as they do great evils to all humanity, and especially to the mothers of our race.

---

For Singers, or for a Bad Cough or Sore Throat.—Take the white of one egg, beat it to a stiff froth; add two teaspoonsful of white sugar; take a teaspoonful frequently until relieved. This is worth any quantity of cough mixtures, as it is eaten with avidity by young children, and does not disorder the stomach. The albumen and the carbon supplied by the egg and sugar are invaluable to persons of weak lungs. This recipe is constantly used by prima donnas before going on the stage to sing, as it wonderfully improves the voice.

